# Community-driven development for computational biology at Sprints, Hackathons and Codefests

**DOI:** 10.1186/1471-2105-15-S14-S7

**Published:** 2014-11-27

**Authors:** Steffen Möller, Enis Afgan, Michael Banck, Raoul JP Bonnal, Timothy Booth, John Chilton, Peter JA Cock, Markus Gumbel, Nomi Harris, Richard Holland, Matúš Kalaš, László Kaján, Eri Kibukawa, David R Powel, Pjotr Prins, Jacqueline Quinn, Olivier Sallou, Francesco Strozzi, Torsten Seemann, Clare Sloggett, Stian Soiland-Reyes, William Spooner, Sascha Steinbiss, Andreas Tille, Anthony J Travis, Roman Valls Guimera, Toshiaki Katayama, Brad A Chapman

**Affiliations:** 1 University of Lübeck, Department of Dermatology, Germany; 2 Debian Project; 3 Center for Computing and Informatics, Ruđer Bošković Institute (RBI), Croatia; 4 VLSCI Life Sciences Computation Centre, University of Melbourne, Australia; 5 Istituto Nazionale Genetica Molecolare "Romeo ed Enrica Invernizzi, Milano, Italy; 6 NERC Centre for Ecology and Hydrology, Wallingford, UK; 7 University of Minnesota Supercomputing Institute, USA; 8 The James Hutton Institute, UK; 9 Mannheim University of Applied Sciences, Germany; 10 Lawrence Berkeley National Laboratory, Berkeley, CA, USA; 11 Eagle Genomics Ltd, Wakefield, MA, USA; 12 now at Pistoia Alliance, Wakefield, MA, USA; 13 University of Bergen, Computational Biology Unit and Department of Informatics, Norway; 14 TU Munich, Department of Bioinformatics and Computational Biology, Germany; 15 Illumina Inc., Japan; 16 Monash University, Victorian Bioinformatics Consortium, Australia; 17 Department of Medical Genetics, Institute for Molecular Medicine, University Medical Centre Utrecht, The Netherlands; 18 Autodesk, Inc., USA; 19 University of Rennes, INRIA/Irisa, France; 20 Parco Tecnologico Padano, Lodi, Italy; 21 University of Manchester, School of Computer Science, UK; 22 University of Hamburg, Center for Bioinformatics, Germany; 23 University of Aberdeen, Institute of Biological and Environmental Sciences, Aberdeen, Scotland, UK; 24 Stockholm University, Department of Biochemistry and Biophysics, Science for Life Laboratory, Sweden; 25 Database Center for Life Science, Tokyo, Japan; 26 Harvard Public School of Health, Boston MA, USA

## Abstract

**Background:**

Computational biology comprises a wide range of technologies and approaches. Multiple technologies can be combined to create more powerful workflows if the individuals contributing the data or providing tools for its interpretation can find mutual understanding and consensus. Much conversation and joint investigation are required in order to identify and implement the best approaches.

Traditionally, scientific conferences feature talks presenting novel technologies or insights, followed up by informal discussions during coffee breaks. In multi-institution collaborations, in order to reach agreement on implementation details or to transfer deeper insights in a technology and practical skills, a representative of one group typically visits the other. However, this does not scale well when the number of technologies or research groups is large.

Conferences have responded to this issue by introducing Birds-of-a-Feather (BoF) sessions, which offer an opportunity for individuals with common interests to intensify their interaction. However, parallel BoF sessions often make it hard for participants to join multiple BoFs and find common ground between the different technologies, and BoFs are generally too short to allow time for participants to program together.

**Results:**

This report summarises our experience with computational biology Codefests, Hackathons and Sprints, which are interactive developer meetings. They are structured to reduce the limitations of traditional scientific meetings described above by strengthening the interaction among peers and letting the participants determine the schedule and topics. These meetings are commonly run as loosely scheduled "unconferences" (self-organized identification of participants and topics for meetings) over at least two days, with early introductory talks to welcome and organize contributors, followed by intensive collaborative coding sessions. We summarise some prominent achievements of those meetings and describe differences in how these are organised, how their audience is addressed, and their outreach to their respective communities.

**Conclusions:**

Hackathons, Codefests and Sprints share a stimulating atmosphere that encourages participants to jointly brainstorm and tackle problems of shared interest in a self-driven proactive environment, as well as providing an opportunity for new participants to get involved in collaborative projects.

## Background

Sprints, Hackathons and Codefests are all names for informal software developer meetings, especially popular in Open Source communities. These meetings, which often take place in loose conjunction with more traditional conferences, are a vital part of the international network of interactions between software developers working in bioinformatics and computational biology, and complement purely online interactions such as project mailing lists, online chat, web forums, voice and video calls.

Collaborative development of software has figured significantly in bioinformatics for over 20 years. Leveraging and building upon existing Open Source software is a powerful way to rapidly implement new ideas and methods into reliable working code. This helps in a world where scientific groups are under increasing pressure to produce results quickly and more cheaply than ever. The challenge for everyone is to be aware of existing implementations of a particular desired functionality and their compatibility with local infrastructure. Strategically, it is beneficial to know other contributors to externally maintained libraries, and to ensure that contributions are integrated with the remaining code in the best future-compatible way and with the least possible redundancies.

This paper summarises the activities and backgrounds of three related types of meetings: Sprints, Hackathons and Codefests. These share the aim of fostering collaborative interactions and the trust to allow mutual dependencies between developers in computational biology and bioinformatics. Although these meetings share common features, each event has its own particular slant and flavour of the community.

## Methods

### Hackathons

*Focus on bringing together existing developers of closely related projects, to accelerate development while encouraging inter-project cohesion*.

A series of BioHackathons (short for "biologically motivated code hacking marathons") have been held. The BioHackathons [1-3] have been organized as an invitational event with the loose intention of encouraging the participants to collaborate on a given theme. This flexibility recognises that with hindsight the most productive results/ideas were not always predictable beforehand, but emerged from self-organized collaborative work during the BioHackathons when developers from different domains spent a week talking and coding together.

The original BioHackathons in 2002 and 2003 were mainly dedicated to interoperability in handling sequence data amongst the Bio* projects. BioPerl, BioJava, Biopython, and BioRuby groups worked together to develop common sequence object models, APIs for the BioSQL database and Web services. This ensured that fundamental bioinformatic functionality would be compatible amongst those four programming toolkits. The first BioHackathon resulted in the BioPerl publication [4].

### Codefests

*Bring together a wide range of developers to find new points of collaboration, encourage integration and spawn new projects*.

The Bioinformatics Open Source Conference (BOSC) was established in 2000 by the Open Bioinformatics Foundation Bio* project members as an international venue for showcasing new projects and progress, and for developers worldwide to meet in person. Since then BOSC has been held yearly as a special interest group (SIG) meeting preceding the annual Intelligent Systems in Molecular Biology (ISMB) conference, one of the most popular bioinformatics conferences. Since 2010 the annual BOSC meetings have also included a two-day informal BOSC Codefest, which has typically attracted between 25 and 50 participants. Meeting in person is a valuable complement to traditional online distributed teamwork, allowing more intensive discussions and social bonding that continues into the BOSC meeting.

Over 30 developers participated in the BOSC 2013 Codefest, hosted by Humboldt-University in Berlin. Projects accomplished by attendees included the extension of several existing open-source tools, development of standards for provenance tracking, and integration of infrastructure management, visualization and parallelization frameworks [5]. A key outcome was increased interoperability between tools, an essential requirement for carrying out large scale science in rapidly evolving research areas [6]. Specific BOSC 2013 Codefest accomplishments included small updates in the Biopython and Cloud Bio-Linux projects, and work on integration of SLURM, an HPC job manager, to the iPython Cluster. Scala is an alternative language to Java that runs in the same JVM environment. During the 2013 Codefest the Scala SCABIO code was integrated into BioJava, with the developers working together to ensure that the result was implemented cleanly and without redundancy.

The BioRuby group tackled a new project which enables programmers to develop Web applications for BaseSpace [http://basespace.illumina.com], a cloud solution provided by Illumina on which users can apply various analysis tools to next generation sequencing (NGS) data. During the 2013 Codefest, a Ruby version of the BaseSpace SDK was tested, documented and completed for release as an Open Source package. Recently, the BaseSpace Ruby SDK was contributed to Illumina as one of the official toolkits along with the Python, Java and R versions with the help of a participant from Illumina.

Mobyle [5] was modified to provide provenance data using W3C PROV-O [6] and integrated with the EDAM ontology [7] for describing its services. The Synthetic Biology Open Language Visual Standard [8] had been formalized as an ontology, and the 2013 Codefest brought together Semantic Web engineers who were consulted on how to model the visual representation of concepts within the ontology.

Sometimes at Codefests existing technologies are retired in favour of new ones. For the last eight years, RNAmmer has been the standard tool for predicting ribosomal RNA features in genomes. Its drawbacks are that it relies on small, old databases; requires an obsolete version of HMMER; and has restrictive licence terms that prevent anyone beyond the authors from distributing improved code. To resolve these issues for prokaryotes, a new rRNA predictor was implemented which uses the new "nhmmer" tool from HMMER 3.1 for searching DNA profiles against DNA sequences. This led to the development of Barrnap [http://www.vicbioinformatics.com/software.barrnap.shtml], which will be packaged in Bio-Linux and replace RNAmmer in the Prokka bacterial annotation system [9]. The identification of the problem, a technical solution and its immediate employment in larger workflows needed several individuals to work together to agree on the approach and begin the required coding--a type of interaction fostered by Codefests and similar events.

### Sprints

*Focus on a single overarching project or technical challenge, providing a meet-up for existing developers as well as a way to involve and mentor new project members*.

When Linux surfaced as a free operating system, it was adopted quickly by the research community, including many bioinformatics developers. This, along with the ethos of open data in the major international biological databases [10], led to a large body of software developed for Linux and released with Free/Open licenses. For example, the Open Source Bio* projects, most prominently BioPerl [4], BioJava [11], Biopython [12] and BioRuby [13,14], originated over 14 years ago as community projects providing widely used libraries for building bioinformatics tools, pipelines and one-off analysis scripts. The situation also led to a natural alliance between bioinformatics software developers and the community-supported Linux distributions they use.

To help promote the redistribution, general availability and mutual compatibility of software, the Debian Linux project launched the Debcamp event, a hacking session right before the Debian Conference. Debcamp is an "unconference", a meeting at which people meet and work on specific topics, either alone or in teams. Along the same lines, the Debian Med initiative was founded in 2001 [15], with the Debian Sprint held as an annual meeting since 2011.

Debian Med [16] and Bio-Linux [17] provide the necessary infrastructure for distributing software tools and their updates to the wider community. This is achieved by packaging and distributing the tools in the context of these larger tool repositories. The Cloud Bio-Linux community in turn further enhances the distribution, tailored for bioinformatics on cloud infrastructure [18]. The Sprint helps with the integration of these efforts.

The 2013 Debian Sprint invited contributors to BOINC [19], a distributed computing project, and we found the binaries of Debian, auto-compiled for multiple different architectures, to be directly usable in BOINC projects across Linux distributions. Together with an ongoing effort to provide packages for the BOINC server side, this will help increase the availability of compute time for biological groups, e.g., for biochemists with a novel receptor addressing protein docking [20]. This is a good example of the positive outcomes that can result from the cross-disciplinary collaborative activities at Sprints and similar events.

The integration of a single software package can trigger a collection of multiple other software tools to be packaged together to complete common workflows in that field. For example, porting of the GenomeTools software suite [21] during the 2012 Debian Med Sprint not only integrated a substantial number of published and established sequence analysis tools into Debian, but also paved the way for future inclusion of packages dependent on the associated GenomeTools library, e.g., ParsEval [22] or LTRsift [23] or the wealth of PredictProtein [24]. This emphasizes the role of Sprints as strong promoters of synergistic effects within the community.

## Results

Having 20+ talented and motivated individuals with shared interests work together for two or more days can be extremely productive and have a major impact on that development community. Each person who participates in these collaborative meetings brings their own complement of technical, scientific and social strengths. These developer meetings allow for cross-pollination of skills between separated silos of expertise. These events can be organised around any topic with a large enough user base. They combine individualised training, social networking and technical contributions and help pave the way toward new scientific discoveries. Results include real software solutions, documentation, and the joint communication of milestones for future developments.

Each event described in this paper has its own culture and organization. The Japanese BioHackathon events are the longest, at one week each, and this series is also the longest running and best-funded, with support for travel of international participants. It has already yielded several journal publications. The Debian Med Sprints are noteworthy for bringing together "upstream" developers (who are directly working on scientific Free Software projects) and "downstream" scientific users and developers (who are working on Debian Med derived distributions), along with core and prospective Debian Med members. This heterogeneous group allows for more efficient skill exchange and a wider array of topics. Everybody can weigh in with their particular strength, while letting the rest of the group focus on their particular interests and strong points.

## Discussion

Since Open Source software developers spread across the globe already collaborate by communicating online via distributed source-code repositories, mailing lists, chat and other means, the time and expense of travelling to meet up in person may seem like a waste. However, physical meetings bring an edge to productivity, including temporarily avoiding day-to-day workplace duties, and the opportunity to see software and infrastructure problems from outside of one's local needs. Also, meeting in person temporarily solves the problems of cross-time-zone collaborations. This is particularly important for contributors in Australasia communicating with Europeans or Americans, where live interactions like conference calls must be often scheduled outside normal office hours, and any conversation by email can take days. Meeting physically also helps build interpersonal relationships and can motivate attendees to follow up on issues they might not tackle otherwise. Most people feel more of a sense of connection and commitment to people they have worked with in person than to those they have never met. It fosters projects that in fact depend on the geophysical distribution of participants, e.g., for the OpenDataDay [http://opendataday.org], and the networking also has a career advantage [25].

**Figure 1:**
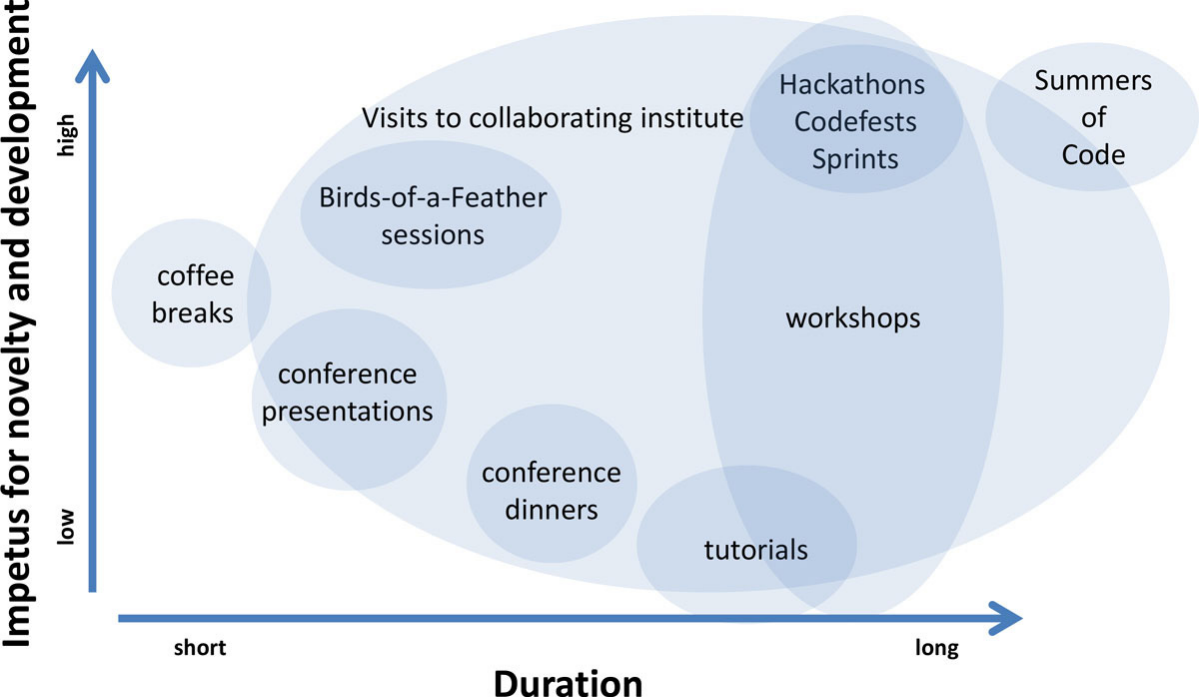
**Forms of academic exchange**. *The most common opportunities for scientists to meet separated by their drive for novelty and development (Y axis) and their duration (X axis), the former as a subjective consensus among the authors. Sprints, Codefests and Hackathons dominate for their focus on joint new developments, the transfer of expertise for new scientific questions, the distribution of infrastructure and a network of trust between the contributors. Longer programs like the Summer of Code combine many types of interactions over a long time, with the difference that those participating may be assigned different, specific roles - say as mentor and learner*.

**Figure 2:**
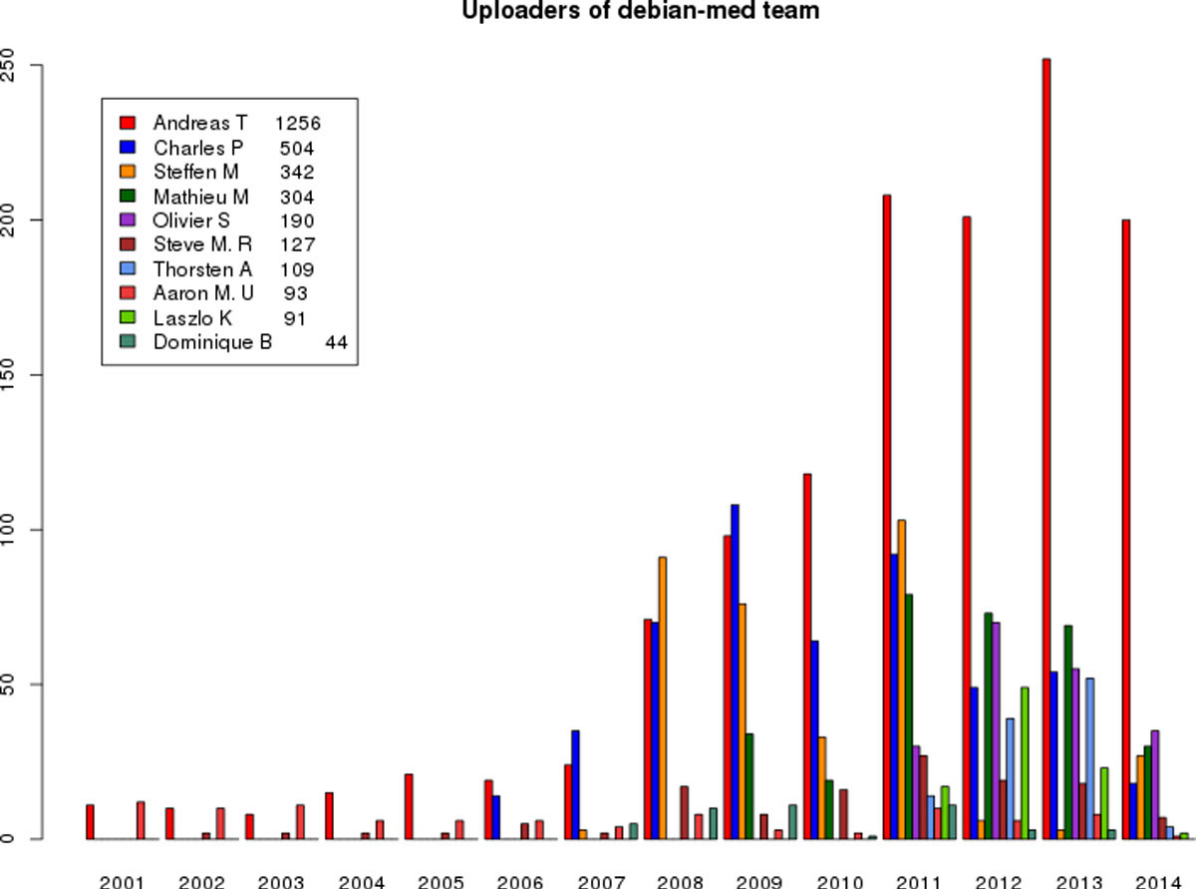
**Number of uploads to Debian Med per individual**. *The figure from *http://blends.debian.net/liststats/ *indicates the activity of team members with upload privileges. One clearly sees the increased breadth since the first Sprint in early 2011.*.

Other frameworks exist to encourage interactions, such as the concept of a Summer of Code (as run, for example, by Google or the European Space Agency), combining remote collaborations with a summit at the end. Many if not most of the contributors to the events we have discussed here also mentor for the Google Summers of Code and find it complementary to the self-organised events - much like extra resources for the researchers' interest and, if attending the summit, an opportunity to look deeply beyond one's personal area of expertise [26]. The events described here focus on the science and easier access to technology in an intense way that is open to everyone (Figure 1). This underscores the Sprints' direct effect on the activity of the projects, both in terms of the number of patches submitted and, on the social side, the number of emails distributed on the mailing list (Figure 2).

A considerable change observable with the advent of Synthetic Biology is that engineers and computer scientists are building and using tools for tasks that have in the past been performed manually by biologists, e.g., planning of cloning experiments. We need to learn to address and attract such neighbouring communities to co-develop and share Open Source infrastructure to avoid being crowded out by closed-source solutions. A critical mass of software solutions and users for the synthetic biology field has yet to emerge, and the interplay between Open Source and commercial entities is yet to be established. The BOSC 2013 Codefest [27] helped establish first contact between the Bioinformatics and Synthetic Biology communities, and hopefully will lead to helpful interchange between the fields.

Not only academic or research institutions use Open Source tools or frameworks. Open Source tools are widely used in commercial research and commercial service providers who build products and services around Open Source components. Both of these types of companies have an interest in improving the quality of the Open Source software they use. Hackathons and Codefests offer an opportunity for these improvements to be made whilst simultaneously meeting with the original developers, learning from them, and giving them guidance as to future requirements.

The BOSC Codefest and the Debian Med Sprint are more constrained as a two-day meeting than the week-long Hackathon. As a consequence, there is less time for the participants to be trained or self-educated within the group, or to pursue larger projects within the meeting itself. Here, the Google Summer of Code, with its months-long individualised training, has an advantage. Larger, more long-term programs of collaboration can be sustained where participants can make a significant commitment in availability, and also benefit from external funding and peer incentives to participate. The smaller events described in this paper require a smaller up-front commitment so that most prospective participants can find the time to attend, and are also run on a shoestring budget so a rich sponsor is not necessary.

The motivation for small companies to get involved with an Open Source Hackathon or Codefest is typically based on the expectation that the participation will have a positive effect on the perception of the companies' products and in anticipation of additional sales **- **whether they are taking part actively or simply sponsoring the event. Large research corporations that use Open Source products internally may want to get involved only if the event will develop features and fix bugs that will improve the company's internal productivity, and hence save operational costs. A service or product provider that uses Open Source tools may only want to participate if the outcome is an improved tool or feature set that they can then build commercial offerings around for their own customers, or use to improve their own internal processes and reduce overheads. To attract greater participation from commercial partners, Hackathons and Codefests must therefore include a certain amount of applied research driven by the requirements of these partners, and be willing to guide their development efforts in a direction that will deliver commercial value. This may not resonate with participants from a pure academic research background where commercial requirements are a much lower priority, but it is essential to gain external sponsorship. For computational biology, the underlying infrastructure is mostly Open Source because of the historic freeness of the sequence data. On the applications side, however, e.g., for the assembly and optimisation of workflows, for which the Assemblathon [28] may stand representatively, or user-centric design [29], the license of widely distributed tools is not of concern.

## Conclusions

Participation in a Hackathon, Codefest, or Sprint can be an extremely rewarding experience for the participants and the greater community. These informal, interactive meetings have played an important historical role in the development of Open Source technologies and are now benefiting the bioinformatics community. At these events, developers of all ages and levels of experience interact with each other. Besides the joint problem-solving work, these events can encourage new contributors to surface. Hands-on training and exchange of experiences, actively and passively, remain core features of the events.

In closing, we can point to specific examples of software developments and bug fixes made during the developer meetings described, and in some cases publications that have resulted from these meetings. However, their true worth is less immediately tangible in the form of the community itself, new and strengthened collaborations, and the spread of ideas and best practices - both scientific and for software development.

## Competing interests

The authors declare that they have no competing interests.

## Authors' contributions

All authors contributed to the preparation of the document and participated in at least one of the described events.

## Declarations

Eagle Genomics and the Lübeck Department of Dermatology shared the fee for this Open Access publication.

This article has been published as part of *BMC Bioinformatics *Volume 15 Supplement 14, 2014: Semantic, social, mobile applications for bioinformatics and biomedical laboratories: selected works from the 13th International Workshop on Network Tools and Applications in Biology (NETTAB 2013). The full contents of the supplement are available online at http://www.biomedcentral.com/bmcbioinformatics/supplements/15/S14.
